# Knowledge, practice and attitude associated with SARS-CoV-2 Delta Variant among adults in Jordan

**DOI:** 10.1371/journal.pone.0278243

**Published:** 2022-12-07

**Authors:** Ghadeer A. R. Y. Suaifan, Ala’ M. Abu-Odeh, Mayadah B. Shehadeh, Rula M. Darwish, Moyad Shahwan, Fahid Abu Jbara

**Affiliations:** 1 Department of Pharmaceutical Sciences, School of Pharmacy, The University of Jordan, Amman, Jordan; 2 Department of Pharmaceutical Chemistry and Pharmacognosy, School of Pharmacy, Applied Science Private University, Amman, Jordan; 3 Department of Pharmaceutics and Pharmaceutical Technology, School of Pharmacy, The University of Jordan, Amman, Jordan; 4 College of Pharmacy and Health Sciences, Ajman University, Ajman, United Arab Emirates; 5 School of Medicine, The University of Jordan, Amman, Jordan; Hospital Universitat de Sant Joan, Universitat Rovira I Virgili, SPAIN

## Abstract

COVID-19 infection is a global pandemic health emergency. This contagious disease was caused by the Severe Acute Respiratory Syndrome Coronavirus‑2 (SARS‑CoV-2) which is mutating over time. In 2021, the Delta variant became the most dominant transmissible form. During the crisis, human practice and knowledge were critical in the overall efforts to encompass the outbreak. A cross-sectional, web-based approach was conducted among adults in Jordan to quantify knowledge, attitude, and practices towards SARS-CoV-2 (Delta variant). This research was carried out between 15^th^ April and 15^th^ of May 2021. The study questionnaire consisted of four sections including the participant’s demographics, knowledge, practices and attitude. Comparative evaluation of responses was accomplished using a scoring system. Respondents who scored above the mean score (60%) on the item measured were categorized as knowledgeable, having a positive attitude, and good practices. Participants were allocated to one of the three groups; medical, non-medical and others (unemployed and housewives). Data collected was analyzed using Statistical Package for Social Sciences (SPSS) version 23.0 software. A variance test to assess the statistical difference between groups was used. Pearson’s chi-squared test was applied to compare the variables and identify significant predictors. Of the participants, 308 (66%) were in the age group of 18-25yrs, 392 (84.1%) females, 120 (25.8%) employed and 346 (74.2%) unemployed. The principle source of knowledge was social media (291, 62.4%). Interestingly, participants had adequate overall knowledge. The mean knowledge score was 22.6 (± 0.19), 20.6 (± 0.19), and 21.3 (± 0.18) for the medical, the non-medical and the others group, respectively. Also, participants showed a positive attitude and good practices towards SARS-CoV-2 (Delta variant). The mean practice score for medical, the non-medical and the others groups was 7.35 (± 0.25), 7.38 (± 0.24), 7.35 (± 0.24) and the mean attitude score was 10.8 (± 0.16), 9.4 (± 0.21), 9.5 (± 0.22), respectively. The studied groups generally had good knowledge, positive attitudes and good practices about SARS-CoV-2 (Delta variant). This was expected due to the authorities’ successful management of the pandemic and the high educational level of the Jordanian society, bearing in mind the economic and social impact of COVID-19 disease.

## Introduction

A global public health concern emerged in 2019 with coronavirus disease (COVID-19). This contagious disease was caused by the Severe Acute Respiratory Syndrome Coronavirus‑2 (SARS‑CoV-2) [1–5], which had no predictable scale of infectivity due to the high rate of infection and prevalence, asymptomatic transmission, long incubation period, genetic modification and seasonal differences [5]. On March 11, 2020, the World Health Organization declared the coronavirus pandemic as a global health emergency concern [2, 6–8].

Currently applied protocols for COVID-19 management includes medications as protease inhibitors (Lopinavir, Ritonavir), RNA polymerase inhibitors (Favipiravir, Remdesivir, Ribavirin), hydroxychloroquine, neuraminidase inhibitors (Oseltamivir), antiviral cytokine (Interferon-β-1a) and convalescent immunoglobulins obtained from recovering patients [7], in addition to vaccination using Sinopharm, AstraZeneca, Pfizer and BioNTech, Moderna-NIAID, and Johnson and Johnson vaccines to retard COVID-19 spread [9]. Although these vaccines can decrease COVID-19 incidence and increase the overall population immunity, their efficacy, safety and availability are still questionable. Thus, implementation of non-pharmaceutical intervention (NPI) aims to lower mortality. Today, the cornerstone for preventing COVID-19 spread is personal hygiene and physical distancing [10–12].

SARS‑CoV-2 consists of four different structural proteins. The mutations in these proteins are the most important in determining the virulence of the virus [13, 14]. The S-protein (spike) of the SARS‑CoV-2 virus is the main part of antibody therapy and vaccines. Therefore, it is exposed to multiple mutations [14, 15]. Evidence suggests that SARS-CoV-2 has mutated over time, and multiple variants spread worldwide. Up to the time of this study, three different variants of coronavirus have emerged; B.1.1.7 alpha variant (United Kingdom variant), B.1.351 beta variant (South African variant), and P.1 gamma variant (Brazilian variant). All are more transmissible than the original SARS-CoV-2 virus [14–16]. Table 1 summarizes SARS-CoV-2 variants characteristics.

**Table 1:** SARS-CoV-2 variants.

SARS-CoV-2 variants	Properties
Alpha	Increased severity and transmissibility
Beta	Increased severity and transmissibility, possible reduction of vaccine effectiveness
Gamma	Increased severity and transmissibility, possible reduction of vaccine effectiveness
Delta	Highly severe and transmissible, possible reduction of vaccine effectiveness

In 2021, the Delta variant became the most dominant variant flowing globally and was identified as being more transmissible strain [17]. Delta variant is two times more contagious, may cause more severe symptoms in unvaccinated subjects, and may reduce the vaccine effectiveness [9]. This mutated variant may mitigate vaccination benefits because they lessen the binding between the S-protein and the antibodies and might augment the binding strength between the S-protein and angiotensin-converting enzyme, increasing SARS-CoV-2 infectivity [18, 19]. A study by He et al., suggested that optimizing vaccination programs to elicit a strong immune response in both systemic circulation and mucosal tissues might be a valuable approach to tackle Delta variant transmission and infection [19].

Globally, different public health NPI were applied by authorities to control the disease transmission and the burden of COVID 19, including wearing a mask in public places, staying home when ill, committing physical distancing, avoiding crowded and high-risk locations and vaccination. In Jordan, a number of studies have covered the use of NPI measures among healthcare subgroups such as physicians, nurses and medical students [20–25]. A study by Khasawneh *et al*., reported that Jordanian medical students had good precautionary measures and implemented proper NPI strategies to prevent the virus spreading [24]. However, recent study be Khatatbeh *et al*., showed that Jordanian population commitment to the NPI measure was not satisfactory, explaining the observed increase in the infectivity rate of COVID-19 in the country [26]. Researchers recommended strict policies to prevent incoming waves of disease transmission by different threat variants in the future [27]. Interestingly, a study by Boutzoukas *et al*., showed that schools implemented universal masking, retained low transmission level of SARS-CoV-2 Delta variant within the school [28].

In Jordan, around 700000 confirmed cases were detected with 9314 deaths (https://covid19.who.int/region/emro/country/jo). To control the SARS‑CoV-2 virus spreading, the Jordanian government-enforced public health infection prevention and control strategies through social distancing, seized all forms of movement or international travel, completed country isolation, and increased awareness about the seriousness and danger of the virus [29].

The current study aims to assess the knowledge, attitude and practice regarding the new emerging Delta variant among adults in Jordan. The outcomes are expected to help authorities to modulate their policies as needed to prevent the spread of the virus.

## Materials and methods

### Study design

This study used a cross-sectional, web-based approach to quantify knowledge, attitude, and practice among medical and non-medical participants toward SARS-CoV-2 (Delta variant). This research was conducted between 15^th^ April and 15^th^ of May 2021. A convenient, affordable and time-efficient sampling method was used to enroll potential participants. The questionnaire was created, structured and analyzed using the Google forms^®^ and was disseminated via multiple social media outlets, including email, WhatsApp and Facebook to adults with access to the online survey (i.e., internet users in Jordan). The language used in the questionnaire was Arabic, the native language of the targeted community. The eligibility criteria were chosen based on acquiring relevant and sufficient data.

A total of 561 individuals were recruited to participate in the study. Inclusion criteria included adults living in Jordan, aged 18 years and above, active social-media users, capable of reading and understanding Arabic and agreed to fill the online form. Additionally, participants were incited to share the questionnaire with their family members, friends and relatives following the chain-referral or snowball sampling technique. Based on the Jordanian Ministry of Health recommendations at the time of this study, online surveys were deemed the most appropriate method for data collection during this pandemic due to self-isolation, and home quarantine protocols applied.

### Sample size calculation

Sample size estimation at 95% significance level and 5% error margin was calculated utilizing OpenEpi, Version and Kish formula15 using the equation below [30]

**Sample size:**

n=[DEFF*Np(1-p)]/[(d2/Z21-α/2*(N-1)+p*(1-p)]

Where,

N: Population size (for finite population correction factor or fpc)

P: Hypothesized % frequency of outcome factor in the population: 5%±5

d: Confidence limits as % of 100 (absolute ±%): 5%

DEFF: Design effect (for cluster surveys): 1

Z: Value for 95% confidence limits

p: Estimated prevalence

Accordingly, the ideal sample size should be 384. However, considering the non-response rate, a10% of the calculated sample size was added. Thus the final representative sample size for this study was calculated to be 422 [30].

### Measures

Data collection for the current study was acquired using a web-based Google forms^®^ survey in Arabic language as a tool. The study tool was piloted on 50 participants to develop and validate. The distributed questionnaire consisted of four sections, the first section reported the participant’s demographics, and the second targeted the participant’s knowledge regarding the SARS-CoV-2 Delta variant including (a) source of knowledge, (b) ways of transmission, (c) risk factors, (d) ways of prevention and (e) treatment. In the third section, participants were asked about their practices (hand washing, mask-wearing, social distancing and behaviors) and in the fourth section, participants were questioned about their attitudes. To control participants’ attention during the questionnaire filling, three quality control check questions were distributed along the questionnaire. The quality control questions were; have you been diagnosed with COVID-19 since the start of the pandemic, have you been infected with COVID-19, and have you had a positive PCR test for COVID-19? The participant who failed to pass the check questions was excluded from the study.

### Data management

Comparative evaluation of participants’ knowledge, attitude and practice was accomplished using a scoring system [31]. One point was given for each correct response and zero point for incorrect answer. Participants with `I do not know answers`were excluded to eliminate inaccuracies. The total knowledge, practice and attitude scores ranged from 0 to 29, 0 to 10, and 0 to 12, respectively (S1 Table in S1 File). Respondents who scored above the mean score (60%) on the item measurement were categorized as being knowledgeable, had good practice, and positive attitudes (Tables 4 and 5 and 6). Participants were allocated to one of the three groups; medical, non-medical and others (unemployed and housewives). The analysis of variance test (ANOVA) was used to assess the statistical difference between the groups. Post- hoc analysis, Tukey’s Honest Significant Difference test, was applied to examine the specific group’s means (compared with each other). Each time, the comparison occurs between two groups (medical/ non-medical, medical/ others, non-medical/ others). The test compares all possible pairs of means. A *p*-value less than 0.05 was considered statistically significant. Differences in the knowledge, attitude and practice levels between different groups across various demographic variables were evaluated by using the Pearson Chi-Square test (Table 7).

### Ethics approval and consent to participate

Ethical approval was obtained from the Institutional Review Board (IRB) at The University of Jordan (IRB 4–2022). No consent was obtained as the data were collected and analyzed anonymously. Information related to the study aim, the voluntary nature of participation, and participants’ right to refuse or withdraw from the study at any point was written at the beginning of the online questionnaire. Upon accepting participation in the study, participants were redirected to the questionnaire.

## Results

### Sample demographic characteristics

A total of 561 participants filled out the online survey between the 15^th^ of April and the 15^th^ of May 2021. Ninety-five forms were removed from the study due to incorrect responses to the quality control checks questions. Demographical details of respondents are presented in Table 2. Overall, 66% of the participants were in the age group of 18–25 yrs. The majority (84.1%) were females and 74.3% were students, housewives and unemployed (Table 2).

**Table 2:** Demographic characteristics of the participants in Jordan (n = 466).

Characteristics	Number	(%)
AGE		
18–25	308	66.1
>25	158	33.9
GENDER		
Female	392	84.1
Male	74	15.9
EDUCATION LEVEL		
High school	52	11.2
College	24	5.2
Bachelor’s degree	360	77.3
Master	24	5.2
PhD	6	1.3
PROFESSION		
Medical Student	169	36.3
Non-medical Student	54	11.6
Medical Personnel	52	11.2
Non-medical Personnel	68	14.6
Housewives and unemployed	123	26.4
MARITAL STATUS		
Single	323	69.3
Married	133	28.5
Divorced	5	1.1
Widowed	5	1.1

### Source of participants’ knowledge

Social media and the Jordan Ministry of Health were the major sources of the participants’ knowledge as shown in Table 3.

**Table 3:** Participant’s source of knowledge.

Source	Medical group n = 221	Non-medical group, n = 122	Others^♦^ n = 123	Total n (%)
Health care providers	140 (63.3%)	42 (34.4%)	44 (35.8%)	226 (48.5%)
Friends/relatives	43 (19.5%)	39 (32.0%)	32 (26.0%)	195 (41.8%)
Television	60 (27.0%)	48 (39.3%)	46 (37.4%)	194 (41.6%)
Social media	130 (58.8%)	84 (68.9%)	77 (62.6%)	291 (62.4%)
Jordan Ministry of Health	145 (65.6%)	79 (64.8%)	62 (50.4%)	286 (61.4%)

^♦^ Others (unemployed and housewives)

### Knowledge of respondents regarding SARS-CoV-2 (Delta variant)

The mean knowledge score was 22.6 (± 0.19), 20.6 (± 0.19), and 21.3 (± 0.18) for the medical, the non-medical and the others groups, respectively based on subjects’ responses to the measures summarized in S1 Table in S1 File. The study showed no significant relationship between participants’ gender (P = 0.246), age (p = 0924), education level (p = 0.516), profession (0.579) and the level of knowledge (Table 7).

### 1- Knowledge regarding SARS-CoV-2 (Delta variant) transmission

Table 4 showed that 96% reported that COVID-19 new strains could be transmitted from human to human, and 75% identified the inhalation of contaminated air as a mode of transmission. The medical group was more aware about SARS-CoV-2 (Delta- variant) transmission via breathing contaminated air compared to the housewives and the unemployed group.

**Table 4:** Participants’ knowledge regarding SARS-CoV-2 (Delta- variant) transmission, risk factors, prevention and treatment.

Question	Medical group		Non-medical group		Others^♦^
	Student (n = 169)	Employee (n = 52)	Student (n = 54)	Employee (n = 68)	(n = 123)
**Knowledge about the way of transmission of SARS-CoV-2 (Delta- variant)**					
Breathing contaminated air **^(0.36)^	131(77.5%)	46 (88.5%)	35 (64.8%)	53 (77.9%)	86 (69.9%)
From animal to human	41 (24.3%)	14 (26.9%)	11 (20.4%)	16 (23.5%)	21 (17.1%)
From human to human	161 (95.3%)	52 (100%)	51 (94.4%)	64 (94.1%)	120 (97.6%)
**Knowledge about the risk factors of SARS-CoV-2 (Delta- variant)**					
Direct physical contact with an infected person is a risk factor	157 (94.1%)	52 (100%)	51 (94.4%)	61 (89.7%)	113 (91.9%)
Sharing personal belongings	153 (90.5%)	49 (94.2%)	47 (87.0%)	57 (83.8%)	112 (91.1%)
Traveling to affected places	152 (89.9%)	51 (98.1%)	51 (94.4%)	56 (82.4%)	107 (87.0%)
Visiting clinics*^(0.00),^**^(0.003)^	141 (83.4%)	45 (86.5%)	35 (64.8%)	43 (63.2%)	84 (68.3%)
Failure to adhere to safety guidelines	155 (91.7%)	48 (92.3%)	42 (64.8%)	61 (89.7%)	109 (88.6%)
Being present in gathering *^(0.00),^***^(0.012)^	142 (84.0%)	45 (86.5%)	31 (57.4%)	48 (70.6%)	98 (79.7%)
Pet owners, pet store workers or veterinarians	88 (52.1%)	31 (59.6%)	24 (44.4%)	30 (44.1%)	57 (46.3%)
Health care workers	165 (97.6%)	52 (100%)	50 (92.6%)	63 (92.6)	117 (95.1%)
Travel to epidemic areas*	165 (97.6%)	52 (100%)	51 (94.4%)	65 (95.6%)	120 (97.6%)
Cardiovascular disease*^(0.012)^	131 (77.5%)	46 (88.5%)	40 (74.1%)	42 (61.8%)	95 (77.2%)
Diabetes *^(0.002),^**^(0.028)^	97 (57.4%)	37 (71.2%)	22 (40.7%)	29 (42.6%)	57 (46.3%)
Chronic lung disease**^(0.038)^	155 (91.7%)	52 (100%)	46 (85.2%)	61 (89.7%)	105 (85.4%)
Cancer*^(0.00)^	103 (60.9%)	41 (78.8%)	22 (40.7%)	30 (44.1%)	67 (54.5%)
Chronic kidney disease*^(0.00)^	100 (59.2%)	39 (75.0%)	19 (35.2%)	31 (48.4%)	65 (52.8%)
Smoking*^(0.00),^**^(0.00)^	122 (72.2%)	38 (73.1%)	26 (48.1%)	34 (50.0%)	51 (41.5%)
**Knowledge about the ways of prevention of SARS-CoV-2 (Delta- variant)**					
People can protect themselves from new the strain	132 (78.1%)	45 (86.5%)	40 (74.0%)	44 (64.7%)	90 (73.2%)
Use of face mask**^(0.022)^	152 (89.9%)	52 (100%)	48 (88.9%)	60 (88.2%)	102 (82.9%)
Hand washing correctly* ^(0.009)^	151 (89.3%)	49 (94.2%)	44 (81.5%)	52 (76.5%)	99 (80.5%)
Rub of hands with alcohol*^(0.005),^**^(0.017)^	142 (84.0%)	40 (76.9%)	43 (79.6%)	39 (57.4%)	84 (68.3%)
Commit to social distancing	156 (92.3%)	51 (98.1%)	50 (92.6%)	61 (89.7%)	113 (91.9%)
Do not touch eyes, nose or mouth*^(0.003),^**^(0.00)^	145 (85.8%)	48 (92.3%)	41 (75.9%)	47 (69.1%)	81 (65.9%)
Cover nose, and mouth when sneezing**^(0.002)^	142 (84.0%)	47 (90.4%)	44 (81.5%)	50 (73.5%)	86 (69.9%)
Stay at home when feeling ill	150 (88.9%)	50 (96.2%)	45 (83.3%)	57 (83.8%)	104 (84.6%)
**Knowledge about effective treatments for SARS-CoV-2 (Delta- variant)**					
Antibiotics	96 (56.1%)	24 (46.2%)	26 (48.1%)	43 (63.2%)	64 (52.0%)
Vitamin D and Zn	135 (79.9%)	46 (88.5%)	46 (85.2%)	61 (89.7%)	108 (87.8%)
Herbs	90 (53.3%)	30 (57.7%)	35 (64.8%)	40 (58.8%)	57 (46.3%)
Aspirin	117 (69.2%)	25 (48.1%)	42 (77.8%)	51 (75.0%)	83 (67.5%)
Analgesics and antipyretics*^(0.00),^***^(0.00)^	134 (79.3%)	47 (90.4%)	26 (48.1%)	42 (61.8%)	100 (81.3%)

* Medical/ non-medical,

** Medical/others,

*** Non-medical/others: ANOVA, Tukey’s tests, p value < 0.05 (significant)

^♦^ **Others (unemployed and housewives)**

### 2-Knowledge regarding SARS- CoV-2 (Delta variant) risk factors

The majority of the participants recognized the risk factors associated with SARS- CoV-2 (Delta variant), including ‘direct contact with infected persons’ (93.1%), ‘sharing personal stuff’ (89.7%), ‘traveling to affected areas’ ‘(89.5%), ‘visiting clinics’ (74.7%), ‘failing to adhere to safety guidelines (89.0%) and ‘being present in gathering’ (78.1%). Generally, medical group were more knowledgeable about high risk patients in comparison with other groups as shown in Table 4.

### 3- Knowledge regarding SARS-CoV-2 (Delta variant) ways of prevention

All groups had a good knowledge about the protective measures against the transmission of Delta variant (Table 4). Approximately 89% of the participants acknowledged the routine use of the face mask, 85% washed their hands correctly, and 75% rubbed their hands with alcohol. In addition, participants were aware of other preventative acts such as social distancing, covering the nose and mouth when sneezing, and staying home when feeling ill. As well, 85.3% of the participants reported the importance of avoiding or touching the nose, eyes, and mouth with unwashed hands. The medical group was more aware than the non-medical group about the importance of hand washing correctly (*p* = 0.009), rubbing hands with alcohol (*p* = 0.005), and not touching eyes, nose or mouth (*p* = 0.003) to prevent the Delta variant from spreading. Whereas, no significant differences between the non-medical and the other groups regarding the major preventive methods was observed (Table 4).

### 4-Knowledge regarding SARS-CoV-2 (Delta- variant) effective treatments

The majority (75.0%) of the participants agreed on the use of over-the-counter analgesics and antipyretics to treat the symptoms in addition to the use of vitamin D and zinc supplements (85.0%). However, 45.7% declared the use of antibiotics against SARS-CoV-2 (Delta- variant), yet half of the participants (54.3%) were aware that antibiotics are effective against bacteria but not viruses. The use of complementary herbs was common among 54.1% of the respondents (Table 4). These results indicated participants’ poor knowledge regarding proper SARA-CoV-2 delta variant treatments.

### Practices of participants related to SARS-CoV-2 (Delta variant)

The mean practice score was 7.35 (± 0.25), 7.38 (± 0.24), and 7.35 (± 0.24) for the medical, the non-medical and the others groups, respectively. Thus, the overall mean practice scores were considered “good” practice for all groups. Among the participants, medical and non-medical employees indicated proactive practices more than students.

In general, participants adhered to World Health Organization (WHO) good practices guidelines to prevent the speed of SARS-CoV-2 Delta variant as only five participants (1.1%) reported poor practices in all questions (Table 5). However, a malpractice regarding the proper hand washing technique recommended by WHO (washing hands with soap and water for at least 20 seconds) as an important measure to prevent virus spreading was observed in three quarters (76.2%) of the participants. Correct hand washing techniques was practiced by 24.4% of the medical, 27.8% of the non-medical and 18.7% of the housewives and unemployed group. In addition, only half of the participants in all groups covered their mouth correctly with hands when sneezing (Table 5). Forty percent of the participants greeted others by hand-shaking or hugging and kissing (n = 182). The correlation coefficient between knowledge and practices was 0.91 (*P* = 0.016), revealing the significant association between them, i.e., those who have a better knowledge of SARS-CoV-2 Delta variant have taken more preventive measures.

**Table 5:** Good practices followed by participants.

Question	Medical group		Non-medical group		Others^♦^
	Student (n = 169)	Employee (n = 52)	Student (n = 54)	Employee (n = 68)	(n = 123)
Washing hands correctly (water & soap for 20 sec.)	41(24.3%)	13 (25.0%)	17 (31.5%)	17 (25%)	23 (18.7%)
Using disposable face mask (Once)	164 (97.0%)	52 (100%)	54 (100%)	68 (100%)	117 (95.1%)
Staying away from person who is coughing	164 (97.0%)	52 (100%)	54 (100%)	68 (100%)	117 (95.1%)
Wearing face mask all time in public places*^(0.008)^	148 (87.6%)	50 (96.2%)	36 (66.7%)	59 (86.8%)	105 (85.4%)
Adhering to social distancing	148 (87.6%)	52 (100%)	43 (79.6%)	64 (94.1%)	108 (87.8%)
Avoiding social events	124 (73.3%)	41 (78.8%)	41 (75.9%)	57 (83.8%)	91 (74.0%)
Sneezing (cover mouth with hands)	73 (43.2%)	26 (50.0%)	29 (53.7%)	29 (42.6%)	72 (58.5%)
Sneezing (cover mouth with tissue)	85(50.3%)	19 (36.5%)	22 (40.7)	28 (41.2%)	42 (34.1%)
Sneezing (cover mouth with face mask)	10 (5.9%)	7 (13.5%)	3 (5.6%)	11 (16.2%)	8 (6.5%)
Greeting friends (maintain safe distance)	98 (58.0%)	38 (73.1%)	21(38.9%)	48 (70.6%)	78 (63.4%)
Taking supplements to boost your immune system	96 (56.8%)	30(57.7%)	36(66.7%)	44 (64.7%)	76 (61.8%)
Washing hands as soon as you get in house	165 (97.6%)	50(96.2%)	48(88.9%)	67 (98.5%)	117 (95.1%)

*Medical/Others,

**Medical/ Others,

***Non-medical/ Others: ANOVA, Tukey’s tests, p value< 0.05 (significant)

^♦^ Others (unemployed and housewives)

### Attitudes of participants toward SARS-CoV-2 (Delta variant)

The mean attitude score was 10.8 (± 0.16), 9.4 (± 0.21), and 9.5 (± 0.22) for the medical, the non-medical and the others groups, respectively. Thus, the overall mean practice scores were considered a “good” attitude for the study subjects. The study revealed a significant correlation between participants’ level of attitude and gender (*P* = 0.007) as well as the profession (*P* = 0.024) as shown in Table 7.

The question with the highest number of negative responses was about the belief of whether the virus was man-made and if it is a kind of biological weapon (Table 6). Surprisingly, this thought was more prevalent in the medical group than in the others. In addition, about half of the participants reported that they found it difficult to report any suspected case to the authorities (Table 6).

**Table 6:** Participants’ attitude.

Question	Medical group		Non-medical group		Others^♦^
	Student (n = 169)	Employee (n = 52)	Student (n = 54)	Employee (n = 68)	(n = 123)
Is a man-made disease* ^(0.01),^ **^(0.002)^	65 (38.5%)	27(51.9%)	17 (31.5%)	14 (20.6%)	21 (17.1%)
Is a biological war	55 (32.5%)	21 (40.4%)	9 (16.7%)	16 (23.5%)	18 (14.6%)
Think that new COVID strains can be prevented**^(0.015)^	155 (91.7%)	45 (86.5%)	40 (74.1%)	61 (89.75)	99 (80.5%)
Quarantine is useful	111 (65.7%)	43 (82.7%)	30 (55.6%)	51 (75.0%)	89 (72.4%)
Advice that you give to someone sick (do PCR)	147 (87.0%)	42 (80.8%)	43 (79.6%)	54 (79.4%)	97 (78.9%)
Acceptance to take vaccine*^(0.02)^	120 (71.0%)	37 (71.2%)	20 (37.0%)	44 (64.7%)	85 (69.1%)
Welling to travel to the area of SARS-CoV-2 (Delta- variant) for work	19 (11.2%)	7 (13.5%)	5 (9.3%)	5 (7.4%)	6 (4.9%)
Traveling to an area of SARS-CoV-2 (Delta- variant) for leisure**^(0.01)^	8 (4.7%)	2 (3.8%)	2 (3.7%)	3 (4.4%)	6 (4.9%)
Steps you do if you come in contact with an infected person: (nothing)	6 (3.6%)	2 (3.8%)	0	2 (2.9%)	7 (5.7%)
You will report any suspected cases	72 (42.6%)	30 (57.7%)	26 (48.1%)	37 (54.4%)	65(52.8%)
Did you feel afraid of your family	141 (83.4%)	47 (90.4%)	40 (74.1%)	56 (82.4%)	88 (71.5%)
Don’t attend funerals	149 (88.2%)	46 (88.5%)	35 (64.8%)	53 (77.9%)	95 (77.2%)
Don’t attend weddings	147 (87.0%)	45 (86.5%)	42 (77.8%)	56 (82.4%)	100 (81.3%)
Don’t attend Friday prayer	141 (83.4%)	44 (84.6%)	32 (59.3%)	43 (63.2%)	80 (65.0%)

*Medical/non-medical,

**Medical/ Others,

***Non-medical/ Others: ANOVA, Tukey’s tests, *p* value< 0.05 (significant)

^♦^ Others (unemployed and housewives)

Overall, participants believed that the new SARS-CoV-2 (Delta variant) is preventable. In addition, 70% of the participants thought quarantine was a helpful method to prevent the spread of new strains; 69.7% for the medical, 66.4% for the non-medical and 72.4% for the others group. Other common positive attitudes among participants includes advising a person who is sick to do PCR (82%) and refrained from attending social events (funerals (81%), weddings (83.7%), and Friday prayer (73%)) (Table 6).

More than half of the participants’ consent to take the vaccine. The acceptance of taking a vaccine was more prevalent in the medical group compared to the non-medical group. However, participants were worried about contracting the infection and transmitting it to family members due to their interaction with the public (Table 6).

## Discussion

In December 2019, a new coronavirus-induced disease (COVID-19) emerged in China, caused by SARS-CoV-2 airborne virus. Globally, exceptional measures were taken to restrict and control COVID-19 outbreak, including cities lockdowns, closure of universities, schools and businesses, pausing traveling, and quarantining defined cases from other healthy people [32].

Due to the prompt spread of SARS-CoV-2, different strategies were drawn to boost public awareness about the seriousness of the disease and to increase public attention to precautionary measures [33]. This cross-sectional study investigated the knowledge, practice and attitude about the new Delta- variant of SARS-CoV-2 among the adults in Jordan. The study sample has a good education level with a high female gender predominance (Table 2). This slanting towards education and female participation was observed in previous studies ran in Jordan [34–36]. In addition, more than half of the respondents were below the age of 25 years (Table 2). Jordan demographics are well-known to be predominated by the younger population [37].

Collected data showed that participants were optimistic regarding the new SARS-CoV-2 Delta variant being not of a major concern. In agreement with AlKhasawneh study [38], social media was a common source of knowledge among the study groups. Furthermore, undergraduate students used the internet, the social media and the mass media as sources to get information about the Delta variant [39]. Likewise, El-Elimat study, indicated that Jordanians trusted health coworkers for information about SARS-CoV-2 [24, 40]. Generally speaking, social media is a positive, rapid and effective way to disseminate information [6, 41].

Most participants were aware of the different risk factors associated with SARS-CoV-2 Delta variant transmission as physical contact and sharing personal belongings (Table 4). On the other hand, the medical group was aware that SARS-CoV-2 infected patients with underlying chronic illnesses, such as diabetes, hypertension, cardiovascular, cancer, kidney and respiratory diseases are at a higher risk of morbidity and mortality, which is in agreement with the AlKhasawneh study [38].

Notably, around 80% of the study population had high level of awareness about the essential preventative procedures to limit the spread of the disease; maintaining social distance, using a face mask, hand washing, covering mouth and nose when sneezing, staying home when feeling ill and not touching mouth or eyes. Furthermore, other hygiene and disinfection approaches were observed, like handwashing before food preparation.

Participants knew that the most susceptible individuals to be infected are health care workers and those who travel to highly affected areas. This high level of knowledge among medical and non-medical students was previously reported [6]. Factors that may contribute to this level of knowledge include the government’s attempts to boost the awareness towards SARS-CoV-2 generally and about the Delta variant specifically through campaigns [42], accessibility of information through different sources and the high educational level of the study participants [35–37, 43–46].

The noticeable malpractice during sneezing and the failure to commit with WHO hand washing technique even among the medical group is worrying (Table 5). A study by Ragusa *et al*., [47] observed that despite what might be expected, adherence to the procedure did not increase between health coworkers. This is not only explained by the problem of technical knowledge of the procedures, but also to educational, psychological and organizational factors.

The medical and non-medical employees were more aware of all good practices than student participants. This finding may be explained by the less reliable sources and poor quality of information students have access to. Besides, students may be psychologically affected by the governmental lockdown and legislations.

Overall, the medical students showed good knowledge, positive attitudes and practices towards the new variant, which is consistent with a previous study [48]. This finding could be attributed to their ability to criticize the information more readily based on the university education.

Although respondents had sufficient knowledge about the disease, they retained vague thoughts about the possible treatment options (Table 4). Half of the respondents believed that antibiotics and aspirin are effective against SARS-CoV-2 (Delta- variant). In fact, both aspirin and antibiotics (especially azithromycin and hydroxychloroquine) were used to manage certain cases of COVID- 19 when advised by specialists. Therefore, participants should be cautioned of using them empirically [49–51].

It is also worth mentioning that the use of prospected immune-boosting supplements and herbs was increased during the COVID-19 pandemic [52, 53]. The study showed that half of the respondents believed that herbal medicine is effective against Delta variant (Table 4). Such findings were reported among the public in the Middle East who believed in the effectiveness of vitamin supplements and herbal remedies as a treatment option for COVID-19 illness [52–54]. In addition, the lack of efficient medications to fight viral infections has directed attention to medicinal plants as a potential supply of effective and successful treatment.

Only one-third of the participants rejected the idea that the virus was manufactured as a weapon for biological war. The belief in conspiracy theory about the biological war was noticed previously among university students in Jordan [55]. This could be attributed to the false information communicated through the media since which lacks editorial revision and fact-checking. Therefore, it is noteworthy that this thought was reported worldwide [37, 49].

The study revealed good practice toward COVID-19 preventative steps (Table 5). The majority of participants in all groups reported wearing a disposable face mask in public places. The medical students were more willing to take vaccines and more committed to mask-wearing all the time in public places than the non-medical students. Further education regarding the necessity for wearing a face mask by the non-medical student is vital.

Participants through the different study groups adhered to social distancing, avoided large gatherings, shaking hands and hugging. These results were consistent with Alzoubi and Elayeh [37, 56] and were supported by a study conducted throughout the Middle Eastern countries included Jordan, Iraq and Palestine, where participants exhibited reasonable protective behaviors [54].

It is well known that hand hygiene is a cornerstone in preventing disease dissemination. However, 64% of participants washed their hands with water only. This might be attributed to our participants’ young age as they tend to challenge risky behavior [57].

As noted, employed individuals and housewives had positive attitude and committed to good practices compared to students (Table 7). Such act might be attributed to subjects`willingness to protect themselves, their families and to reduce disease transmission.

**Table 7:** Knowledge, practice and attitude level differences between group participants by demographic characteristic.

Variable	Level of knowledge		*p* value	Level of practice		*p* value	Level of attitude		*p* value
	Good	Poor		Good	Poor		Good	Poor	
Female	358 (76.8%)	34 (7.3%)	0.246	380 (81.5%)	12 (2.6%)	0.611	315 (67.6%)	77 (16.5%)	0.007
Male	70 (15.0%)	4 (0.90%)		72 (15.5%)	2 (0.40)		49 (10.5%)	25 (5.4%)	
18–25	283 (60.7%)	25 (5.4%)	0.924	294 (63.1%)	14 (3.0%)	0.060	236 (50.6%)	72 (15.5%)	0.503
26–45	90 (19.3%)	7 (1.5%)		97 (20.8%)	0		76 (16.3%)	21 (4.5%)	
≥ 46	55 (11.8%)	6 (1.3%)		61 (13.1%)	0		52 (11.2%)	9 (1.9%)	
High school	45 (9.7%)	7 (1.5%)	0.516	50 (10.7%)	2 (0.43)	0.484	39 (8.4%)	13 (2.8%)	0.215
College	23 (4.9%)	1 (0.20%)		22 (4.7%)	2 (0.43)		22 (4.7%)	2 (0.43%)	
BSc degree	333 (71.5%)	27 (5.8%)		350 (75.1%)	10 (2.1%)		282 (60.5%)	78 (16.7%)	
Masters	22 (4.7%)	2 (0.40%)		24 (5.2%)	0		18 (3.9%)	6 (1.3%)	
PhD	5 (1.1%)	1 (0.20%)		6 (1.3%)	0		3 (0.64)	3 (0.64%)	
Medical employee	206 (44.2%)	15 (3.2%)	0.579	216 (46.4%)	5 (1.1%)	0.364	183 (39.3%)	38 (8.2%)	0.024
Non-medical employee	111 (23.8%)	11 (2.4%)		119 (25.5%)	3 (0.60%)		88 (18.9%)	35 (7.5%)	
Unemployed	111 (23.8%)	12 (2.6%)		117 (25.1%)	6 (1.3%)		93 (20%)	29 (6.2%)	

*p* value< 0.05 (significant)

WHO has highlighted the significance of taking actions that improve the protection and control of disease transmission in the community. However, our study identified some gaps in the knowledge, practices and attitudes among the different groups towards the SARS-CoV-2 delta variant.

### Limitations of the study

The findings of this study should be interpreted by focusing on these limitations:

The sample may be under-representative as sampling method using social media may bias the study; deprive individuals who can’t deal with or have no access to social media and modern devices. Participants with high education levels and females were over-represented compared to the general population in Jordan. Like other self-reported surveys, the participants’ answers may reflect positive desirability but not the actual situation. The study tool included a number self-reporting practices where subjects may not be able to assess themselves accurately and so report socially acceptable answer rather than being truthful.

## Conclusion

This study showed that participants generally possess basic information and positive attitudes concerning SARS-CoV-2 Delta variant accompanied with proactive practices towards the disease. This can be explained by the global warning of the disease’s seriousness, the authorities’ successful management of the pandemic, and the high educational level in Jordan. Yet, there is a need to improve communication with students regarding adherence to personal protective measures and the importance of vaccination.

## Supporting information

S1 File S1 Table–Knowledge, practice assessment scoring system. (DOCX)

S2 File Supplementary file. (ZIP)
