# Supplementary material for: Knowledge, practice and attitude associated with SARS-CoV-2 Delta Variant among adults in Jordan
Source: PLoS One. 2022 Dec 7;17(12):e0278243. doi: 10.1371/journal.pone.0278243 (PMC9728918; doi:10.1371/journal.pone.0278243)
Supplement: S1 File — (DOCX) [file pone.0278243.s001.docx]

Knowledge, Practice and Attitude associated with SARS-CoV-2 Delta Variant Among Adults in Jordan

Ghadeer A.R.Y. Suaifan^1^*, Ala’ M. Abu-Odeh^2^, Mayadah B. Shehadeh^1,^ Rula M. Darwish^3^, Moyad Shahwan^4^, Fahid Abu Jbara^5^

^1^ Department of Pharmaceutical Sciences, School of Pharmacy, The University of Jordan, Amman 11942, Jordan.

^2^ Department of Pharmaceutical Chemistry and Pharmacognosy, School of Pharmacy, Applied Science Private University, Jordan, Amman.

^3^ Department of Pharmaceutics and Pharmaceutical Technology, School of Pharmacy, The University of Jordan, Amman 11942, Jordan

^4^ College of Pharmacy and Health Sciences, Ajman University, United Arab Emirates

^5^ School of Medicine, The University of Jordan, Amman, Jordan

***Corresponding author**

**Prof. Ghadeer Suaifan**

E-mail: [gh.suaifan@ju.edu.jo](mailto:gh.suaifan@ju.edu.jo)

E-mail: Ghadeer_petra@yahoo.com

**Table 1. Knowledge, Practice Assessment scoring system**

| **Domain** | **Assessment Question** | | | **Number** |
| --- | --- | --- | --- | --- |
| **Knowledge**  **(K)** | How many SARS-CoV-2 variants emerged up to date? | | | **K1** |
|  | What do you think of the following statements about SARS-CoV-2 (Delta- variant)? | Appear to spread more easily and quickly | | **K2** |
|  |  | Transmitted in the same way as the original virus | | **K3** |
|  |  | People can protect themselves from SARS-CoV-2 (Delta- variant) | | **K4** |
|  | Do you think that SARS-CoV-2 (Delta- variant) is more harmful than the original virus? | | | **K5** |
|  | Do you think COVID-19 vaccine is effective against SARS-CoV-2 (Delta- variant)? | | | **K6** |
|  | Individuals who are most at risk of contracting SARS-CoV-2 (Delta- variant) | Pet owners or pet store workers and veterinarians | | **K7** |
|  |  | Health care workers | | **K8** |
|  |  | Travelers to the affected areas | | **K9** |
|  | How are SARS-CoV-2 (Delta- variant) transmitted? | Breath in virus-contaminated air | | **K10** |
|  |  | From animal to human | | **K11** |
|  |  | From human to human | | **K12** |
|  | How to limit the spread of SARS-CoV-2 (Delta- variant) in your community? | Use of the face Mask | | **K13** |
|  |  | Hand washing with soap and water | | **K14** |
|  |  | Rub hands with alcohol-based antiseptics | | **K15** |
|  |  | Commitment to social distancing | | **K16** |
|  |  | Do not touch eyes, nose, or mouth | | **K17** |
|  |  | Cover nose and mouth with the elbow or tissue when coughing or sneezing | | **K18** |
|  |  | Staying home when feeling ill | | **K19** |
|  | Which of the following can be used to treat SARS-CoV-2 (Delta- variant) symptoms? | Antibiotics | | **K20** |
|  |  | Nutritional supplements) vitamins such as (vitamin D, vitamin C and zinc | | **K21** |
|  |  | Medical Herbs | | **K22** |
|  |  | Aspirin | | **K23** |
|  |  | Analgesics and antipyretics | | **K24** |
| **Practice**  **(P)** | Which of the following habits do you practice? | Wash hands with water and soap for 20 sec. | | **P1** |
|  |  | Never reuse disposable face mask | | **P 2** |
|  |  | Stay away from the person who is coughing or sneezing | | **P 3** |
|  |  | Wear a face mask all the time in public places | | **P 4** |
|  |  | Adhere to social distancing | | **P 5** |
|  | Did you intentionally avoid a social event due to the emergence of SARS-CoV-2 (Delta- variant)? | | | **P 6** |
|  | What do you do when you sneeze? | | | **P 7** |
|  | How do you greet your friends/relatives since the emergence SARS-CoV-2 (Delta- variant)? | | | **P 8** |
|  | Do you wash your hands as soon as you get to your house? | | | **P 9** |
|  | Do you take supplements to boost the immune system? | | | **P10** |
| **Attitude**  **(A)** | Do you think quarantine is a good idea to prevent the spread of SARS-CoV-2 (Delta- variant)? | | | **A1** |
|  |  |  |  |  |
|  | Are you willing to travel to an area where SARS-CoV-2 (Delta- variant) has emerged for……. | | Work | **A2** |
|  |  |  |  |  |
|  |  |  |  |  |
|  |  |  | Leisure | **A3** |
|  |  |  |  |  |
|  |  |  |  |  |
|  | What steps would you take if you met individual diagnosed with COVID-19 infection? | | | **A4** |
|  |  |  |  |  |
|  |  |  |  |  |
|  | Will you report any suspected cases of COVID-19 infection? | | | **A5** |
|  |  |  |  |  |
|  | Do you attend some social gatherings despite the emergence of SARS-CoV-2 (Delta- variant)? | | Weddings | **A 6** |
|  |  |  | Funerals | **A 7** |
|  |  |  | Friday praying | **A8** |
